# 

**Published:** 1904-03-26

**Authors:** 


### Publications.

### CHARTS.

**PRICES (Post or Carriage Paid):—**

1,000, 25s. 500, 13s. 6d.; 250, 7s. 6d.; 100, 3s. 6d.; 50, 2s.; 25, 1s. 3d.

SPECIMENS POST FREE.

**THE BEST AND CHEAPEST PUBLISHED.**

GOULD'S TEMPERATURE CHARTS, Morning and Evening.
GOULD'S FOUR-HOUR CHARTS, Printed in Violet for hours 2, 6, 16.
GOULD'S FOUR-HOUR CHARTS, Printed in Chocolate for hours 4, 8, 12.
GOULD'S TWO-HOUR CHARTS.
BENTON'S DIET CHARTS, each Chart lasting two days.
BOARDS to hold Charts when in use, 9d. each, 8s. 6d. per dozen.
PRIVATE NURSING CHARTS, lasting 48 hours.
CHARTS for taking the Temperature of Sick Rooms.

USED IN ALMOST ALL THE PRINCIPAL HOSPITALS AND INFIRMARIES IN THE UNITED KINGDOM.

**WODDERSPOON & CO., 5 Gate Street Lincoln's Inn Fields, W.C.**

ENLARGED. EIGHTH EDITION. Crown 8vo. 10s. 6d.

### PHARMACY, MATERIA MEDICA, AND THERAPEUTICS

is now rewritten and printed in large type, and is made a companion to the following volume. It contains an account of the action **of every drug in the B.P.**, as well as a section upon all the newest drugs, with an Introduction to the Art of Dispensing. With woodcuts, prescriptions, &c.

Also FOURTH EDITION. 1055 pages. 8vo. 16s.

### DICTIONARY OF TREATMENT.

Containing an up-to-date Article upon the Treatment of every Diseased Condition, whether Medical, Surgical, Gynæcological, or Ophthalmological, as well as Articles upon Poisoning, Drowning, and the various Surgical Emergencies and Accidents, arranged for Rapid Reference.

By W. WHITLA, M.A., M.D., Professor of Materia Medica, Queen's College, Belfast; Senior Physician to the Royal Victoria Hospital, &c.

The Publisher will supply both volumes, carriage paid, for **21/-**.

London: HENRY RENSHAW, 356 Strand.

Crown 8vo. Illustrated. Price **1/-** net.

THE

### RADICAL CURE OF CORNS AND BUNIONS.

By E. HARDING FREELAND, F.R.C.S.,

Surgeon to the St. George's and St. James's Dispensary, &c.

"Mr. Freeland has done good service by affording, through proper and professional means, good prospects of relief from the troublesome and often disabling effects of these minor ailments."—*Brit. Med. Jour.*

London: BALE, SONS & DANIELSSON, 83 Gt. Titchfield Street, W.

Crown 8vo. Illustrated. Price **2s. 6d.** net.

### ANÆSTHETICS. By J. BLUMFELD,

M.D.Cantab., Senior Anæsthetist to St. George's Hospital; Anæsthetist to the Grosvenor Hospital for Women and Children, London, and to the National Dental and Metropolitan Hospitals.

*Lancet.*—"....In writing this handbook the author has been quite successful." *Australasian Medical Gazette.*—"We can strongly commend this book." *Medical Chronicle.*—"The book is one which we can heartily recommend to senior students and practitioners."

London: BAILLIÈRE, TINDALL & COX, 8 Henrietta St., Covent Garden.

*JUST PUBLISHED.*

### The Sanatorium Treatment of Consumption.

By T. N. KELYNACK, M.D., M.R.C.P.

Crown 8vo., limp cloth covers, **6d.** net, **7d.** post free.

*Of all Booksellers, or of*

THE SCIENTIFIC PRESS, Ltd., 28 & 29 Southampton Street, Strand, London, W.C.

### THOMAS BRADFORD & CO.

MANUFACTURERS OF

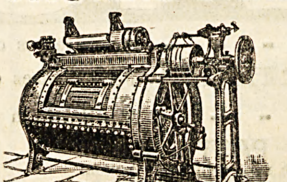

**LAUNDRY MACHINERY**

For Hand, Steam, and Electric Power.

NEW ILLUSTRATED CATALOGUE POST FREE.

LONDON—LIVERPOOL—MANCHESTER

### IMPORTANT NOTICE.

**On account of the Easter Holidays, we beg to announce that we shall go to press with our issue of the 2nd APRIL on TUESDAY NIGHT, the 29th MARCH. Advertisements intended for insertion in this issue should therefore reach us by 4 p.m. ON TUESDAY, the 29th MARCH.**

### NOTICE TO CORRESPONDENTS.

All MSS., Letters, Books for Review, and other matters intended for the Editor should be addressed The Editor, "The Hospital," The Hospital Buildings, 28 & 29 Southampton Street, Strand, W.C.

The Editor cannot undertake to return rejected MS., even when accompanied by stamped directed envelope.

### Notice to Advertisers and Subscribers.

All Advertisements, Orders for Copies of The Hospital, and Business Communications should be addressed to **THE MANAGER (Not to the Editor),** The Hospital, 28 & 29 Southampton Street, Strand, London, W.C.

The Cost of Subscription, post free, is as follows:—Per week, 1½d.; per quarter, 3s. 9d.; per half-year, 7s. 6d.; per annum, 15s. Foreign and Colonial:—Per annum, 19s. 6d., payable, in all cases, in advance.

**NOTICE.—Vol. XXXIV. of "The Hospital" (April 1903 to September 1903), in handsome cloth binding, is now on sale at the office of this Journal, price 10s. 6d. Cases for binding the Numbers contained in this Volume, 2s. Index to Vol. XXXIV., 2½d., post free.**

**The Monthly part for March is now ready. Price 1s., by post, 1s. 3d.**

### Scraps and Gleanings.

It has been decided to erect a bronze statue of the late Mr. Lecky in the precincts of Trinity College, Dublin, as a memorial to the great historian.

The Goldsmiths' Company have made a special grant of £750 to the Royal London Ophthalmic Hospital in response to their centenary appeal. £500 has also been granted to the Metropolitan Convalescent Institution by the same Company.

It is stated that since its formation, 21 years ago, the East End Emigration Fund has assisted 7,000 people to emigrate to British Colonies. The large majority of these have done very well in their new environment. It costs on an average £30 to enable each family to emigrate, and £1,000 are needed to help the families now waiting to go. An appeal is therefore being made by the Society for an increase of subscriptions to their funds.

The Royal Statistical Society is anxious to induce the Government to pass an Act enabling the Registrar-General to take a general census of the country in 1906, instead of waiting the usual ten years. It further suggests that as the enumeration would be intermediate between the two decennial censuses, it might be of a less complicated nature than these, affording information merely as to numbers, sex, and age.

### The Student of Medicine.

### APPOINTMENTS.

**Wilfrid Allport**, M.B., B.S.Lond., M.R.C.S., L.R.C.P., Honorary Ophthalmic Surgeon to the Warneford Hospital, Leamington. **Donald Armour**, M.B., M.R.C.P.Lond., F.R.C.S.Eng., Surgeon in charge of the Nose, Throat, and Ear Department, Belgrave Hospital for Children. **A. C. Dornford**, M.R.C.S., L.S.A., District Medical Officer of the Poplar Union. **A. J. Edmonds**, M.B., Assistant Medical Officer to the Bethnal Green Parish Infirmary. **Percy Flemming**, B.S., F.R.C.S., Professor of Ophthalmic Medicine and Surgery in University College, and Surgeon to the Ophthalmic Department of University College Hospital. **J. Fullarton**, M.B., M.S.Glasg., District Medical Officer of the Lancaster Union. **H. S. Gabb**, M.B., B.C.Cantab., Certifying Factory Surgeon for the Hastings District, Sussex. **Harry Gardner**, M.R.C.S., L.R.C.P., Junior House-Surgeon to the Croydon General Hospital. **T. Herbert Goodman**, M.R.C.S.Eng., L.S.A., Medical Officer and Public Vaccinator for the Haverhill District of the Risbridge Union. **James De Burgh Griffith**, M.B., Ch.M.Dub., Physician to Out-patients, Perth Public Hospital, West Australia. **Otto Grunbaum**, M.B., B.C.Cantab., D.Sc.Lond., M.R.C.P., Assistant Physician to the Belgrave Hospital for Children. **Thomas Houston**, M.D.R.U.I., Joint Lecturer on Medical Jurisprudence in the Queen's College, Belfast. **D. Barty King**, M.A., M.D., M.R.C.P.Lond., Honorary Physician to Queen Alexandra's Home for Officers' Widows and Daughters. **F. H. Lawson**, M.R.C.S., L.R.C.P.Lond., Certifying Factory Surgeon for the Steyning District, Sussex. **Charles J. Marsh**, L.R.C.P., M.R.C.S., Medical Referee under the Workmen's Compensation Act for the Crewkerne, Wincanton, and Yeovil Districts in County Court Circuit No. 35. **Cyril William Milner**, M.R.C.S.Eng., L.R.C.P.Lond., Honorary Consulting Surgeon to the Nottingham General Dispensary. **H. H. Sanguinetti**, M.B., B.Ch.Oxon., Medical Officer of Health for the Minehead Urban District. **W. C. B. Smith**, M.R.C.S., L.R.C.P., Senior House-Surgeon to the Croydon General Hospital. **Henry Martyn Stumbles**, M.B., Ch.B.Edin., Police-Surgeon to the Amble District of the Northumberland County Constabulary. **David Wield**, M.A., M.D.Edin., Honorary Assistant Gynæcologist at Brisbane General Hospital. **H. B. Woodcock**, M.B., B.Ch.Vict., District Medical Officer of the Chorlton Union.

**"The Hospital" Institutional Library.**

"Hospitals and Asylums of the World." 4 Vols., with a Portfolio of Plans, complete £12 12s.

"Burdett's Hospitals and Charities, 1903." 5s. net.

"Burdett's Official Nursing Directory, 1903." 8s. net.

"Cottage Hospitals, General, Fever, and Convalescent." 10s. 6d.

"Uniform System of Accounts for Hospitals and Public Institutions." New Edition. 4s. net.

"Hospital Expenditure: The Commissariat." 2s. 6d.

"A Legal Handbook for the Use of Hospital Authorities." 2s. 6d.

"The Cottage Hospital Case Book or Register of Patients." 10s. and 12s. 6d.

All these are published by the Scientific Press, Ltd., and may be obtained through any bookseller, or direct from the publishers, 28 and 29 Southampton Street, Strand, London, W.C.

